# Profil épidémiologique des tumeurs malignes primitives des glandes salivaires : à propos de 154 cas

**DOI:** 10.11604/pamj.2014.17.117.2939

**Published:** 2014-02-18

**Authors:** Khadija Setti, Mohamed Mouanis, Abdelmounim Moumni, Mostafa Maher, Amal Harmouch

**Affiliations:** 1 Laboratoire d'Anatomie Pathologique, Hôpital des Spécialités, Faculté de Médecine, Université Mohamed V Souissi, Rabat, Maroc

**Keywords:** Epidémiologie, tumeurs malignes, glandes salivaires, Epidemiology, malignant tumors, salivary glands

## Abstract

**Introduction:**

Les tumeurs des glandes salivaires sont des tumeurs rares représentant 3à 5% des tumeurs de la tête et du cou. La classification de l'OMS 2005 distingue les tumeurs épithéliales, les tumeurs mésenchymateuses, les tumeurs hématologiques et les tumeurs secondaires.

**Méthodes:**

Notre travail consiste en une étude rétrospective réalisée sur une période de 10 ans allant de janvier 2002 à janvier 2012. Les critères d'inclusion étaient: l'âge, le sexe, le siège de la tumeur et le type histologique.

**Résultats:**

L'incidence annuelle des tumeurs malignes primitives des glandes salivaires dans notre série était de 15 cas par an. Cent cinquante quatre cas de tumeurs malignes primitives des glandes salivaires ont été colligés sans prédominance de sexe (78 femmes (50,6%) et 76 hommes (49,4%)). La moyenne d'âge était de 60 ans avec des extrêmes de 4 et 83 ans et un pic de fréquence entre 51et 70 ans. Deux tiers des cas (65%) avaient une localisation au niveau des glandes principales avec 66 cas au niveau de la parotide (43%) et 34 cas au niveau de la glande sous maxillaire (22%). Cinquante quatre patients avaient une tumeur maligne des glandes salivaires accessoires (35%) dont 61% au niveau du palais. Aucun cas de tumeur maligne de la glande sublinguale n'a été recensé dans notre étude. Le type histologique prédominant dans notre série était le carcinome adénoïde kystique et retrouvé chez 43 patients (27,9%), suivi de l'adénocarcinome sans autre indication chez 37 patients (24%) puis du carcinome mucoépidermoïde chez 16 patients (10,4%) et de l'adénocarcinome polymorphe de bas grade également chez 16 patients (10. 4%).

**Conclusion:**

Les tumeurs malignes des glandes salivaires représentent un ensemble hétérogène de maladies de caractérisation complexe et de fréquence variable.

## Introduction

Les tumeurs des glandes salivaires sont des tumeurs rares représentant moins de 1% de toutes les tumeurs du corps et 3à 5% des tumeurs de la tête et du cou. Les tumeurs des glandes salivaires se répartissent selon la dernière classification de l'OMS 2005 en tumeurs primitives épithéliales (dont 2 malignes et 24 bénignes), en tumeurs mésenchymateuses, en tumeurs hématologiques et en tumeurs secondaires. Ces tumeurs sont essentiellement bénignes (60 à 80%). La fréquence des tumeurs malignes primitives varie selon la localisation [1]. Le but de notre travail est de rapporter, à travers une étude rétrospective, le profil épidémiologique des tumeurs malignes primitives des glandes salivaires en les comparants aux données de la littérature.

## Méthodes

Notre travail consiste en une étude rétrospective réalisée dans le service d'anatomie pathologie de l'hôpital des spécialités de Rabat sur une période de 10 ans allant de janvier 2002 à janvier 2012. Les critères d'inclusion étaient : L'âge, le sexe, le siège de la tumeur (glande salivaire principale ou accessoire) et le type histologique. Dans la plupart des cas, il s'agissait de biopsies où ni la taille ni l'aspect macroscopique n'étaient précisés. Par conséquent ces deux critères n'ont pu être exploités.

## Résultats

**Figure 1:**
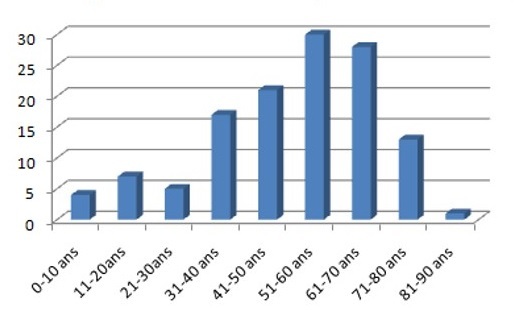
Répartition des cas par tranche d’âge

L'incidence annuelle des tumeurs malignes primitives des glandes salivaires dans notre série était de 15 cas par an. Cent cinquante quatre cas de tumeurs malignes primitives des glandes salivaires ont été colligés sans prédominance de sexe (78 femmes (50,6%) et 76 hommes (49,4%)). La moyenne d'âge était de 60 ans avec des extrêmes de 4 et 83 ans et un pic de fréquence entre 51et 70 ans (Figure 1).

**Figure 2:**
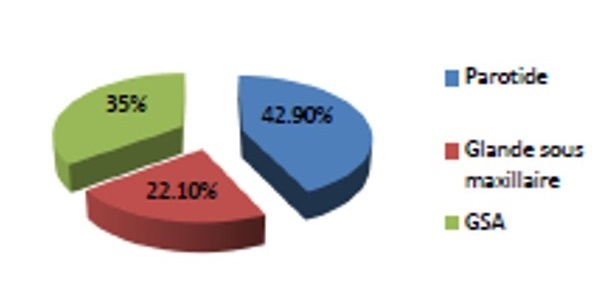
Répartition de toutes les tumeurs selon la localisation

**Figure 3:**
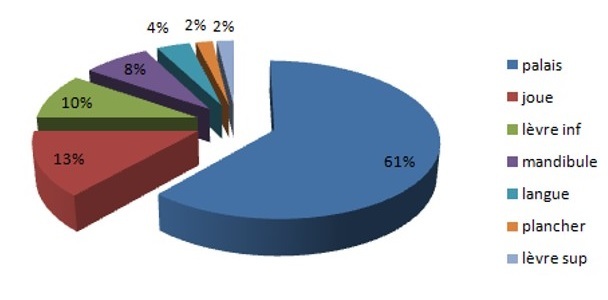
Répartition des tumeurs des glandes salivaires accessoires selon la localisation

Deux tiers des cas (65%) avaient une localisation au niveau des glandes principales avec 66 cas au niveau de la parotide (42,9%) et 34 cas au niveau de la glande sous maxillaire (22,1%) (Figure 2). Cinquante quatre patients avaient une tumeur maligne des glandes salivaires accessoires (35%) dont 61% au niveau du palais (Figure 3). Aucun cas de tumeur maligne de la glande sublinguale n'a été recensé dans notre étude.

**Figure 4:**
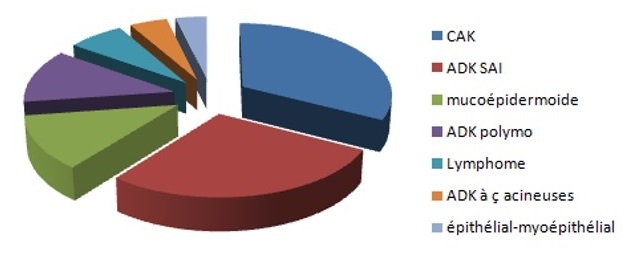
Répartition des types histologiques les plus fréquents

Le type histologique prédominant dans notre série était le carcinome adénoïde kystique (CAK) retrouvé chez 43 patients (27,9%), suivi de l'adénocarcinome sans autre indication (ADK SAI ) chez 37 patients (24%) puis du carcinome mucoépidermoïde chez 16 patients (10,4%) et de l'adénocarcinome polymorphe de bas grade également chez 16 patients (10. 4%) (Figure 4).

Chez les patients âgés de moins de 10 ans, quatre tumeurs ont été retrouvées réparties entre deux CAK, un ADK SAI et un rhabdomyosarcome (RMS) embryonnaire. Concernant la tranche d'âge de 11 à 20 ans, nous avons retrouvé 7 cas dont 2 CAK, 2 RMS embryonnaires, un carcinome mucoépidermoïde, un mélanome et un RMS alvéolaire. Entre 21et 30 ans, les 5 tumeurs retrouvées étaient réparties entre 3 ADK SAI, un lymphome et un carcinome à cellules acineuses. Concernant les patients âgés de 31 à 40 ans, nous avons répertoriés 17 cas dont 14 CAK et 3 lymphomes. Entre 41 à 50 ans, 21 cas ont été retrouvés répartis entre 8 CAK, 5 ADK SAI, 5 ADK polymorphes de bas grade et 3 carcinomes mucoépidermoïdes. Entre 51 et 60 ans, 30 cas ont été retrouvés répartis entre le CAK (8cas), l'ADK SAI (8 cas), le carcinome mucoépidermoïde (7 cas) et l'ADK polymorphe de bas grade (7cas). Dans la tranche d'âge de 61 à 70 ans, 28 cas ont été retrouvés dont les types prédominants étaient le CAK (9cas), l'ADK SAI (7cas), le carcinome mucoépidermoïde (5 cas) et l'ADK polymorphe (4 cas). Chez les patients âgés de 71 à 80 ans, les 12 cas retrouvés étaient des ADK SAI. Le seul patient retrouvé dans la tranche d'âge de plus de 80 ans présentait un ADK SAI. Enfin, l'âge n'a pu être précisé chez 29 patients.

## Discussion

L'incidence annuelle des tumeurs malignes primitives des glandes salivaires dans notre série est de 15 cas par an. Elle est de 4 cas,2 cas et 1 cas par an dans des séries brésiliennes [2], iraniennes [3] et tunisiennes [4] respectivement. Cette incidence élevée dans notre série s'explique par le recrutement spécialisé de notre service en pathologie ORL.

**Figure 5:**
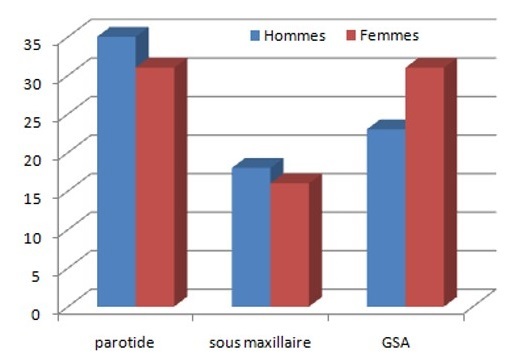
Répartition des cas selon la localisation et le sexe

La moyenne d'âge dans notre série est de 60 ans se rapprochant de celle de la littérature [1]. Dans notre série, les tumeurs intéressent principalement les glandes salivaires principales (65% des cas) et touchent essentiellement les hommes ce qui concorde parfaitement avec les données de la littérature. Par contre les GSA sont rarement touchées dans la littérature alors qu'elles représentent 35 % des cas dans notre série [1, 5]. Ces tumeurs malignes des GSA se voient plus fréquemment chez la femme aussi bien dans notre série que dans la littérature (Figure 5).

### Répartition des différents types histologiques

**Figure 6:**
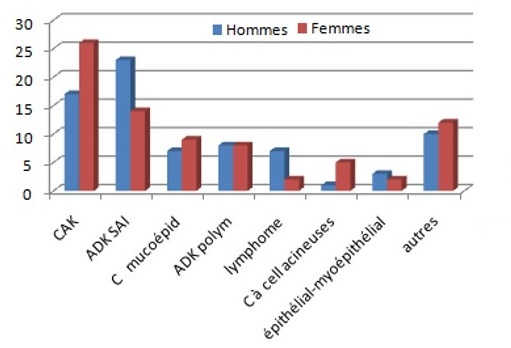
Répartition des types histologiques selon le sexe

**Selon le sexe:** Dans notre série, les types histologiques les plus fréquents chez l'homme sont l'ADK SAI et le lymphome, alors que chez la femme, on note une prédominance du CAK, du carcinome à cellules acineuses et du carcinome mucoépidermoïde (Figure 6).

**Figure 7:**
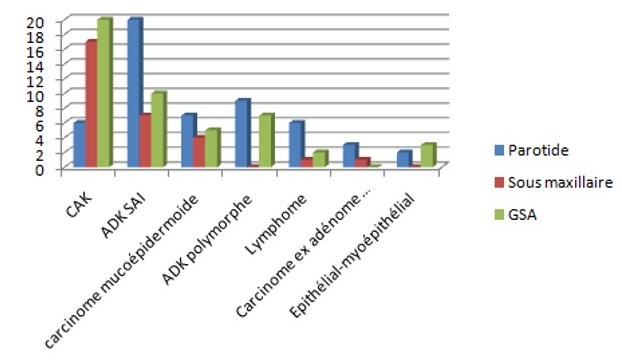
Répartition des différents types histologiques selon la localisation

**Selon la localisation:** Dans notre série, la majorité des tumeurs se localisent au niveau de la parotide (66 cas) répartis entre 17 types histologiques différents. L'ADK SAI étant le type le plus représenté avec 20 cas (30,3%) (Figure 7). Les GSA sont le siège de 54 tumeurs réparties entre 10 types histologiques dont le plus fréquent est le carcinome adénoïde kystique avec 12 cas (22,2%). Ces tumeurs été localisées essentiellement au niveau du palais (32cas /54) . La localisation au niveau de la glande sous maxillaire occupe la 3ème troisième place avec 34cas répartis entre 10 types histologiques dont le plus fréquent est le CAK qui représente la moitié des cas. Dans la littérature consultée, le carcinome mucoépidermoïde représente la tumeur maligne la plus fréquente, alors que dans notre série, les types histologiques prédominants sont le carcinome adénoïde kystique suivi de l'adénocarcinome sans autre indication puis du carcinome mucoépidermoïde.

### Types histologiques

**Carcinome adénoïde kystique:** [1, 6] Le carcinome adénoïde kystique, tumeur maligne très agressive, se voit essentiellement chez la femme entre 40 et 60 ans. Il atteint préférentiellement les GSA et siège souvent au niveau du palais ce qui concorde avec nos résultats.

**Adénocarcinome SAI:** [1, 5, 7, 8] L'adénocarcinome SAI se dé'nit comme étant une tumeur maligne, souvent parotidienne qui ne présente aucune des caractéristiques histologiques des autres types de carcinomes des glandes salivaires. Il s'agit d'une tumeur qui se voit essentiellement chez la femme avec une moyenne d'âge de 58ans. Dans notre série, nous avons retrouvé 37 cas d'ADK SAI dont 23 chez des patients de sexe masculin et 14 patientes de sexe féminin avec un sex-ratio de 16. La moyenne d'âge de nos patients était de 54,2 ans. Cette tumeur se localise préférentiellement au niveau de la glande parotide (60%) puis au niveau des GSA (5-29%) et de la glande sous maxillaire (4%). Dans notre série, ces tumeurs se localisent dans 54% au niveau de la glande parotide, dans 27% au niveau des GSA principalement le palais et dans 19% des cas au niveau de la glande sous maxillaire.

**Carcinome mucoépidermoïde:** Le carcinome mucoépidermoide est la tumeur maligne la plus fréquente de la glande parotide, fréquemment rencontrée à un âge jeune avec une moyenne d'âge de 45 ans et une prédominance féminine. Selon l'OMS et la littérature consultée, Il s'agit de la tumeur maligne la plus fréquente des glandes salivaires (15% à 52%) alors que dans notre série, le pourcentage de carcinome mucoépidermoïde reste faible (10,4%) (Tableau 1). Histologiquement, cette tumeur peut avoir une architecture kystique ou solide et est composée de trois types cellulaires :les cellules mucineuses, les cellules malpighiennes et les cellules de type intermédiaire [1, 6]. La proportion de ces trois types cellulaires varie d'une tumeur à l'autre mais aussi au sein d'une même tumeur.

**Tableau 1:** Fréquence du carcinome mucoépidermoïde

Séries	Pourcentage du carcinoma mucoépidermoïde
Vargas et al (brésil) [6]	52%
Satko et al (Slovaquie) [11]	20%
Masanja et al (Tanzanie) [12]	22,9%
Hashemi et al (Iran) [7]	30%
Moatemri et al (Tunisie) [5]	15,4%
Notre série (Maroc)	**10,4%**

Le pourcentage de cellules malpighiennes peut être faible dans certaines tumeurs, leur mise en évidence nécessite l'utilisation de cytokératines de haut poids moléculaire (CK1, 5, 6, 10, 14). Dans certains cas, le recours à la biologie moléculaire permet de mettre en évidence une translocation particulière t (11 :19)(q21 ;p13) spécifique de la lésion [9]. Le pronostic est habituellement favorable.

**Adénocarcinome polymorphe de bas grade** [10]: Il s'agit d'une tumeur des glandes salivaires accessoires qui se voit plus fréquemment chez la femme avec une moyenne d'âge de 59ans (52 ans dans notre série). Elle se localise le plus souvent au niveau du palais (65%) alors que dans notre série, 56,3% de ces tumeurs se localisent au niveau de la parotide.

**Carcinome à cellules acineuses** [1, 6, 11]: Auparavant surnommée tumeur à cellules acineuses, cette tumeur ayant montré un véritable potentiel de malignité est dénommée actuellement carcinome à cellules acineuses. Cette tumeur se voit à tout âge avec une prédilection pour le sexe féminin. Dans notre série, nous rapportons 6 cas de carcinome à cellules acineuses dont 4 siègent au niveau de la glande parotide, un cas au niveau du palais et un cas au niveau de la lèvre inférieure. Ces données concordent avec les données de la littérature puisque la localisation parotidienne se voit dans 80% des cas

**Carcinome épithélial'myoépithélial:** Il s'agit d'une tumeur maligne rare qui représente moins de 1% des tumeurs malignes [1]. Elle forme de façon caractéristique des structures canalaires composées d'une couche interne de cellules épithéliales et d'une couche externe de cellules myoépithéliales claires. Il s'agit d'une tumeur habituellement de bas grade avec une survie à dix ans supérieure à 70 % et une évolution métastatique (ganglions régionaux, poumon, foie) inférieure à 15 % [1, 5]. Ce carcinome affecte surtout la parotide (60%) et survient le plus souvent chez la femme entre 60 et 70 ans [1]. Dans notre série, nous rapportons 5cas de carcinome épithélial myoépithélial avec une moyenne d'âge de 63,5ans et une localisation parotidienne prédominante concordant avec les données mondiales.

**Carcinome myoépithélial:** Le carcinome myoépithélial est une tumeur maligne exceptionnelle composée exclusivement de cordons de cellules myoépithéliales [1, 6]. Il s'agit d'une tumeur localement très agressive avec des récidives locales fréquentes. Il se localise dans 75% des cas au niveau de la glande parotide et touche les sujets de 55ans en moyenne sans prédominance de sexe. Nous rapportons 3 cas de carcinome myoépithélial (1,9%) dont deux au niveau de la glande sous maxillaire et un au niveau de la parotide.

**Carcinome sur adénome pléomorphe** [1, 5, 12]: Dans notre série, 4 cas sont retrouvés chez des patients de sexe masculin avec une moyenne d'âge de 60 ans ce qui concorde exactement avec les données de la littérature. Le diagnostic nécessite soit l'identification d'un contingent d'adénome pléomorphe bénin associé à un contingent épithélial malin, soit une histoire antérieure de résection d'un adénome pléomorphe dans le même site. Dans notre série des foyers d'adénome pléomorphe typique au voisinage du carcinome ont été retrouvés dans tous les cas.

**Adénocarcinome à cellules basales:** Cette tumeur se caractérise par une morphologie basaloïde des cellules tumorales d'allure bénigne. Le diagnostic de malignité repose sur la présence d'un envahissement tumoral des structures adjacentes [1–6]. Il touche le sujet adulte avec une moyenne d'âge de 60 ans sans prédominance de sexe et se localise dans 90% des cas au niveau de la glande parotide. Dans notre série, nous rapportons un seul cas de carcinome à cellules basales localisé au niveau de la glande parotide chez une patiente de 69 ans ce qui concorde avec les données de la littérature.

**Carcinome canal salivaire:** Le carcinome canalaire salivaire est une tumeur maligne rare mais qui constitue l'entité la plus agressive des tumeurs des glandes salivaires. Il s'agit d'une tumeur de haut grade qui se caractérise par sa parenté morphologique avec le carcinome canalaire in'ltrant du sein. Il se voit essentiellement chez l'homme de 55-65ans et se localise le plus souvent au niveau de la glande parotide et de la glande sous maxillaire, mais exceptionnellement au niveau des GSA [13]. Dans notre série, nous rapportons 2 cas de carcinome canalaire dont un au niveau de la glande parotide chez une femme de 35 ans et un cas au niveau de la glande sous maxillaire chez un homme de 78ans.

**Carcinome à cellules claires SAI (sans autre indication):** Il s'agit d'une tumeur rare de la glande parotide composée exclusivement de cellules claires. Seule une cinquantaine de cas ont été rapportés dans la littérature [5, 14]. Nous n'en rapportons aucun cas dans notre série.

**Carcinome oncocytaire** [5, 6]: Les carcinomes oncocytaires sont rares (moins de 1% des tumeurs des glandes salivaires). Ils sont fréquemment observés en association avec un oncocytome préexistant et se distinguent de ce dernier par une architecture de type adénocarcinomateuse associée à des atypies cytonucléaires. Nous n'en rapportons aucun cas dans notre série.

**Tumeurs des tissus mous:** Les tumeurs des tissus mous représentent 2 à 5% des tumeurs des glandes salivaires et peuvent être bénignes ou malignes. Les sarcomes incluent par ordre de fréquence, l'hémangiopéricytome, le schwannome malin, le 'brosarcome, l'histiocytome 'breux malin, le rhabdomyosarcome, l'angiosarcome et le synovialosarcome. Dans notre série, nous rapportons 4 cas de rhabdomyosarcome (2,6%). Les autres types de sarcomes n'ont pas été retrouvés.

**Tumeurs hématologiques:** Les lymphomes représentent 2 % des tumeurs des glandes salivaires et se localisent essentiellement dans la parotide. Ils atteignent essentiellement la femme entre 60et 70ans. Dans notre série, les patients étaient moins jeunes (49 ans en moyenne) et plutôt de sexe masculin avec un sex-ratio de 3,5/1. Il s'agit le plus fréquemment de lymphomes non hodgkiniens à grandes cellules de phénotype B. Parmi les lymphomes à petites cellules, le plus fréquent est le lymphome B de la zone marginale extra ganglionnaire de type MALT suivi du lymphome du manteau. La sialadénite lymphoépithéliale, associée au syndrome de Sjögren, est considérée comme une lésion précurseur du lymphome de type MALT.

## Conclusion

Les tumeurs malignes des glandes salivaires représentent un ensemble hétérogène de maladies de caractérisation complexe et de fréquence variable. Nous pensons que l'épidémiologie de ces tumeurs au Maroc rejoint celles des autres pays non africains.
